# Core belief challenge moderated the relationship between posttraumatic growth and adolescent academic burnout in Wenchuan area during the COVID-19 pandemic

**DOI:** 10.3389/fpsyg.2022.1005176

**Published:** 2022-09-28

**Authors:** Zhengyu Zeng, Xiaogang Wang, Qiuyan Chen, Yushi Gou, Xiaojiao Yuan

**Affiliations:** ^1^School of Education and Psychology, Southwest Minzu University, Chengdu, China; ^2^Key Research Institute of Humanities and Social Sciences of State Ethnic Affairs Commission, Southwest Minzu University, Chengdu, China

**Keywords:** posttraumatic growth, academic burnout, core belief challenge, adolescent, COVID-19

## Abstract

This study investigates the characteristics of posttraumatic growth (PTG) and academic burnout among adolescents in an ethnic minority area in China during the COVID-19 pandemic, and examines the moderating role of core belief challenge on the association between PTG and academic burnout. This study surveyed 941 secondary school students in Wenchuan using the posttraumatic growth inventory, adolescent academic burnout inventory, core beliefs inventory, and a self-designed demographic questionnaire. The results showed that: (1) Five months after the COVID-19 outbreak in China, the level of PTG among adolescents in the Wenchuan area was high and its prevalence was 32.3%. Among them, the dimension of perceived changes in self was the highest, and the PTG level of junior high school students was higher than that of high school students. The academic burnout level of adolescents, particularly emotional exhaustion, was also high. The academic burnout level of the high school students was higher than that of junior high school students. (2) There was a significant negative correlation between PTG and academic burnout among adolescents. (3) Core belief challenge moderated the relationship between PTG and academic burnout. PTG had a significant negative predictive effect on adolescents’ academic burnout only when the core belief challenge was above a moderate level. These results showed the uniqueness of PTG and psychological behavioral problems of adolescents in ethnic minority areas during the COVID-19 pandemic. They also emphasized the key role of core belief challenge in the process of PTG in reducing adolescents’ academic burnout. Based on these results, recommendations are provided to alleviate academic burnout among adolescents in Wenchuan against the background of the COVID-19 pandemic. While providing full play to ethnic minority cultural characteristics to promote posttraumatic growth, guiding adolescents’ value reflection and cognitive reconstruction should be focused on.

## Introduction

The coronavirus disease (COVID-19) pandemic is a major public health emergency worldwide. Since its outbreak, individuals have experienced many problems such as the rapid transmission of the virus, suspension of school and work, interpersonal isolation, resource shortages, and economic crises, which seriously threatened their physical and mental health (Li et al., 2020; Dozois, 2021; Zheng et al., 2021). In China, the rapid and effective control measures of the Chinese government effectively controlled individuals’ objective exposure to the personal experience of loss and casualties in regions other than key pandemic areas, such as Wuhan; however, subjective trauma exposure remained relatively serious. Subjective trauma exposure refers to subjective fear, with indirect and alternative characteristics, and may lead to post-traumatic stress disorder (PTSD) and various psychological and behavioral problems (Zhang et al., 2021). A systematic review of studies conducted in eight countries, including China, showed a high prevalence of psychological problems among the general population during the outbreak, including PTSD (7–53.8%), depression (4.6–48.3%), anxiety (6.33–50.9%), and stress (8.1–81.9%) (Xiong et al., 2020). As a stress-susceptible population, adolescents have been significantly affected by the COVID-19 pandemic. In addition to the anxiety, fear, and stress caused by the pandemic itself, prolonged home isolation, online learning, and parent–child conflict further exacerbated the adaptation problems of adolescent groups (Guessoum et al., 2020; Wang C. et al., 2020; Wang G. et al., 2020; Zhou et al., 2020; Li et al., 2021).

## Literature review

### Posttraumatic growth

While stressful events can lead to PTSD and various psychological and behavioral problems, it can also lead to positive individual growth. Conceptually, posttraumatic growth (PTG) refers to positive psychological changes experienced because of struggling with a highly challenging event or crisis (Tedeschi and Calhoun, 1996). These positive changes involve diverse fields, including self-change as perceived by individuals, interpersonal relations, and the philosophy of life (Tedeschi and Calhoun, 1996, 2004). Physical diseases, stress events, natural disasters, and seeing the vicarious trauma of others may trigger PTG in individuals. PTG levels are usually influenced by personality (Mattson et al., 2018), social support (Yang et al., 2010) and coping strategies (Kesimci et al., 2005; Schroevers and Teo, 2008), among others. A meta-analysis of trauma subjects, including firefighters, cancer patients, earthquake survivors, and only child-lost parents, showed that the prevalence of PTG after trauma events was between 10% and 77.3% (Wu et al., 2019).

In the context of the COVID-19 pandemic, subjective/objective trauma exposure may also trigger individual PTG. For example, individuals who experience isolation can learn to overcome adversity, improve themselves, and achieve self-transcendence (Zhang et al., 2020). Research has shown that during the COVID-19 pandemic, the prevalence of adolescent PTG was 27.9% among Greek adolescents (Ulset and Soest, 2022) and 22.0% in Hubei Province, a high-risk area in China (Zhen and Zhou, 2021). The incidence of PTG among adolescents in medium-risk areas such as Leshan City and Jianyang City in Sichuan Province was 20.6% (Jian et al., 2022). Overall, girls have significantly higher PTG levels than boys (Erman, 2021; Kristo, 2021). There was significantly lower PTG levels in higher grades than lower grades (Zhen et al., 2022).

### Academic burnout

Burnout is a state of physical and mental exhaustion caused by an individual’s failure to cope successfully with excessive demands of their external resources and energy (Freudenberger, 1974). Academic burnout refers to students’ emotional exhaustion due to excessive learning needs, alienation, and indifference to learning, and their sense of worthlessness and low sense of achievement in school (Schaufeli et al., 2002). Academic burnout is the most significant manifestation of burnout among teenagers. The stimulus–response interaction model of stress shows that stressful events may lead to burnout (Carson, 2001). Studies on adolescents who experienced tornadoes (Yuan et al., 2018) and earthquakes (Zhou et al., 2017) have found that stress trauma can significantly predict academic burnout levels.

The COVID-19 pandemic has been widespread and long-lasting. Its repeated uncertainty and closed environment leads to a mismatch between the needs and available resources of adolescents, making them prone to academic burnout (Salmela-Aro and Upadyaya, 2014). A study of college students in Shandong Province, China, showed that 39.29% of students had a high degree of academic burnout during the COVID-19 pandemic (Wang et al., 2021). Studies in Croatia, the United States, and Poland have also found varying degrees of academic burnout among students (Zis et al., 2021; Žuljević et al., 2021; Tomaszek and Muchacka-Cymerman, 2022). Overall, freshmen and graduates have higher levels of academic burnout (Gonzalez-Ramirez et al., 2021). This is also significantly higher among high school students than junior high school students (Sunawan et al., 2021). There are no significant gender differences (Li and Li, 2015), although girls’ emotional exhaustion is approximately twice as high as that of boys (Aljadani et al., 2021).

### Relationship between PTG and academic burnout

PTG has a positive effect on alleviating post-traumatic psychological and behavioral problems. Many studies have shown that PTG helps alleviate psychological problems, such as anxiety (Milam et al., 2005), depression (Vaughn et al., 2009), emotional distress (Ickovics et al., 2006), and burnout among social workers (Gibbons et al., 2011). However, existing research on adolescent academic burnout is rare and inconsistent. Researches on middle school students after the Yancheng tornado showed that PTG had a significant negative effect on academic burnout (Li Y., 2019; An et al., 2022). However, a study of 828 adolescents who experienced the Wenchuan earthquake found that PTG was not significantly related to academic burnout (Lin et al., 2013). This finding suggests that there may be important moderating factors between PTG and academic burnout. For example, Ying et al. (2016) found that trait resilience moderates the relationship between them.

Based on theoretical analysis, this study believes that core belief challenge may play a significant role in the relationship between them. Core belief challenge refers to trauma events that challenge individuals’ prior core belief systems, forcing them to seriously examine each core belief (Janoff-Bulman, 2006). According to the affective-cognitive processing model (Joseph et al., 2012), PTG is an iterative process of growth through event cognition, evaluation mechanism, emotional state, and coping style. The broken assumptive worlds hypothesis (Janoff-Bulman, 1989) highlights that cognitive imbalance after the challenge of core beliefs forces individuals to change their original cognitive style, re-evaluate traumatic situations, and think about their impact. This is more likely to lead to positive behavioral changes. Therefore, a higher core belief challenge may be an important condition for triggering cognitive reappraisal and value reflection, and translating the higher psychological function of individual PTG into action.

### Social and cultural background of Wenchuan area in China

As a minority area in Southwest China, Wenchuan has a unique social and cultural background. First, as a mixed ethnic settlement of Tibetan, Qiang, and Han ethnic groups, the Wenchuan region integrates multicultural and religious beliefs. The local inhabitants are largely influenced by Tibetan Buddhism and traditional Qiang folk beliefs (Ran, 2011; Li, 2019b). Tibetan Buddhism emphasizes an open-minded view of life and death, optimism, conservatism, and gregariousness, to enable individuals to view traumatic events more peacefully (Lan, 2011). The mountain culture of the Qiang people and their awe of nature and obedience to fate can help individuals reduce their post-disaster denial and misfortune (Fan et al., 2009).

Second, during the COVID-19 pandemic, specific pandemic prevention and control measures were implemented in the Wenchuan region. Limited traffic and low population mobility are conducive to preventing and controlling local pandemic, reducing the risk of transmission. Thus, the public’s objective exposure to the pandemic was low. However, economic development in minority areas is relatively regressive and public health services are relatively weak (Fang, 2021). Furthermore, boarding students returned home after schools were suspended. Based on poor online learning conditions, adolescents perceived low support and security during the pandemic; thus, their subjective trauma exposure was relatively high. Based on this social and cultural background, adolescents in Wenchuan may have unique psychological and behavioral responses that differ from those in other regions.

### The current study

It can be seen from the literature review that studies on the characteristics of adolescent PTG and academic burnout in the context of COVID-19 pandemic mainly focuses on mainstream cultural areas, and has not yet seen research on adolescents in ethnic areas. Furthermore, previous studies on the relationship between PTG and academic burnout have focused on natural disaster backgrounds such as earthquakes, tornado, and tsunamis, and the research results are inconsistent. Therefore, this study intends to focus on the following two aspects: (1) Taking adolescents in ethnic minority areas as participants, reveal the characteristics of PTG and academic burnout of adolescents in non-mainstream cultural areas with unique religious culture and social environment during the COVID-19 pandemic. (2) On the basis of theoretical analysis, explore the moderating role of core belief challenge, so as to reveal the relationship between PTG and academic burnout more accurately.

The specific research hypotheses are as follows:H1: During the COVID-19 pandemic, the level of PTG and academic burnout of adolescents in the Wenchuan area is high. There are significant ethnic, gender, and academic stage differences in PTG, and significant ethnic and academic stage differences in academic burnout.H2: During the COVID-19 pandemic, there is a significant negative correlation between PTG and academic burnout among adolescents in the Wenchuan area.H3: During the COVID-19 pandemic, core belief challenge moderates the relationship between PTG and academic burnout in adolescents.

## Materials and methods

### Participants

This study used a cluster sample of 982 adolescents from a secondary school in Wenchuan County, Sichuan Province, China. Questionnaires completed in too short a time were excluded. No participant was excluded because of mental health problems. A total of 941 valid questionnaires (effective rate: 95.82%) were included in the study. The participants included 326 males (34.64%) and 615 females (65.36%), with 238 junior high school students (25.29%), and 703 senior high school students (74.71%). Participants belonged to the following ethnic groups: 435 Qiang (46.23%), 356 Tibetan (37.83%), 110 Han (11.69%), and 40 were from other minorities (4.25%). The average age was 15.95 ± 1.95 years. The Chinese version of the questionnaire was used in this study, because the school curriculum is taught in Chinese and participants can read and write well in Chinese.

### Instruments

#### Posttraumatic growth inventory

The Posttraumatic Growth Inventory developed by Tedeschi and Calhoun (1996) was revised by Zhou et al. (2014). The condition provided in the guidelines was changed to “Since the outbreak of the COVID-19 pandemic.” The revised version has three subscales with 22 items: perceived changes in self, a changed sense of relationships with others, and a changed philosophy of life. Each item is scored on a 6-point scale ranging from 0 (no change) to 5 (a very high degree of change). An example of an item is “I would rather try to change things that need to change.” High total average scores indicated high PTG levels. The PTGI has been used among adolescents in the ethnic minority areas of China with good reliability and validity (Zhou et al., 2015). Cronbach’s alpha was 0.97 in the current study.

#### Adolescent academic burnout inventory

The Adolescent Academic Burnout Inventory by Hu and Dai (2007) was used to measure academic burnout of adolescents during the COVID-19 pandemic. It has four subscales with 21 items: emotional exhaustion, physical exhaustion, alienation between teachers and students, and academic inefficiency. Each item was rated on a 5-point Likert scale ranging from 1 (never) to 5 (always). An example of an item is: “Learning makes me feel uncomfortable.” Higher total average scores indicate higher academic burnout. The inventory has been used among adolescents in the ethnic minority areas of China with good reliability and validity (Zhou et al., 2019). Cronbach’s alpha was 0.90 in this study.

#### Core beliefs inventory

Challenges to core beliefs were measured using a modified version of the Core Beliefs Inventory (Zhou et al., 2014) developed by Cann et al. (2010) and Zhou et al. (2014). The condition provided in the guidelines was changed to “Since the outbreak of the COVID-19 pandemic.” There are nine items, each scored on a 6-point scale ranging from 0 (not at all) to 5 (to a very high degree). A higher total average score indicated a greater degree of challenge to core beliefs. An example of an item is: “After this, I will think about my value as a person.” The inventory has been used among adolescents in the ethnic minority areas of China with good reliability and validity (Zhou et al., 2015). Cronbach’s alpha was 0.95 in the present study.

#### Self-made demographic questionnaire

A self-designed demographic questionnaire was used to collect participants’ demographic information, including age, sex, ethnicity, grade, and pandemic exposure.

### Procedure

We collected online data for this study from April 29, 2020 to May 13, 2020, approximately five months after the outbreak of COVID-19 in China, and one and a half months before the final examinations of the spring semester. Due to pandemic prevention and control requirements, all schools were suspended for months and students attended classes online at home. School leaders, students, and parents provided informed consent. The mental health teacher used a push notification to instruct students to respond to the questionnaire online.

### Data analysis

We used SPSS 26.0 to analyze the data. Variance analysis and independent samples t-tests were used to compare the differences between PTG and academic burnout among demographic variables, such as ethnicity, gender, and academic stage. We conducted a repeated-measures analysis of variance to compare the differences between PTG and academic burnout across the various dimensions. A summary independent samples t-test was conducted to compare our findings with those of previous studies. Pearson’s correlation coefficient was used to calculate PTG, academic burnout, and core belief challenge. Hierarchical multiple regression was used to test the moderating effect of core belief challenge. If the results showed an obvious interaction effect, a simple effect analysis was conducted.

## Results

### Characteristics of PTG among adolescents in Wenchuan area

Table 1 shows the PTG results among adolescents in Wenchuan.

**Table 1:** PTG among adolescents in Wenchuan area during the COVID-19 pandemic (M ± SD).

	Perceived changes in self	A changed sense of relationship with others	A changed philosophy of life	Total average score
Han nationality (*n* = 110)	2.46 ± 1.38	2.36 ± 1.35	2.13 ± 1.21	2.34 ± 1.28
Qiang ethnic minority (*n* = 435)	2.43 ± 1.26	2.40 ± 1.27	2.14 ± 1.10	2.34 ± 1.17
Tibetan (*n* = 356)	2.62 ± 1.26	2.58 ± 1.26	2.37 ± 1.19	2.54 ± 1.19
Male (*n* = 326)	2.60 ± 1.33	2.54 ± 1.33	2.29 ± 1.20	2.49 ± 1.25
Female (*n* = 615)	2.47 ± 1.26	2.44 ± 1.26	2.21 ± 1.14	2.39 ± 1.17
Junior high (*n* = 238)	2.72 ± 1.33	2.69 ± 1.32	2.38 ± 1.19	2.62 ± 1.23
Senior high (*n* = 703)	2.44 ± 1.26	2.40 ± 1.26	2.19 ± 1.15	2.36 ± 1.18
Total	2.51 ± 1.28	2.48 ± 1.28	2.24 ± 1.16	2.43 ± 1.20

Average mean scores >3 on the PTGI indicate moderate levels of PTG (Tang, 2006; Xu and Liao, 2011). In this study, 32.3% of the adolescents reported moderate or high levels of PTG. Assuming a similar degree of harm in the COVID-19 pandemic and using the same survey tools, the study of 2,090 secondary school students in Leshan City and Jianyang City, Sichuan Province, was used as a reference (Tang et al., 2022). A summary independent samples t-test was conducted on the total average score and PTG standard deviation. The results showed that the PTG level of adolescents in Wenchuan was significantly higher than that of secondary school students in Leshan and Jianyang, Sichuan Province [*t* (3029) = −13.56, *p <* 0.001].

Single factor analysis of variance and independent samples t-tests were used to test the ethnic, gender, and academic stage differences in PTG. The results showed no significant differences in ethnicity [*F* (2, 898) = 2.97, *p* > 0.05] and gender [*t* (939) = 0.50, *p* > 0.05] in adolescents with PTG during the COVID-19 pandemic. Furthermore, the PTG of junior high school students was significantly higher than that of senior high school students [*t* (939) = 2.88, *p* < 0.01].

A repeated-measures analysis of variance was conducted to compare the three dimensions of the PTG. The results showed significant differences in the scores among the dimensions [*F* (1.64, 1880) = 1308.52, *p* < 0.001]. Post-hoc comparisons indicated that the dimension of perceived changes in self was significantly higher than in the other two dimensions (*p* < 0.05), and the changed sense of relationship with others was significantly higher than the changed philosophy of life (*p* < 0.001).

### Characteristics of academic burnout among adolescents in Wenchuan area

Table 2 shows the results of adolescents’ academic burnout in Wenchuan.

**Table 2:** Academic burnout among adolescents in Wenchuan area during the COVID-19 pandemic (M ± SD).

	Emotional exhaustion	Alienation between teachers and students	Academic inefficiency	Physical exhaustion	Total average score
Han nationality (*n* = 110)	2.29 ± 0.69	1.97 ± 0.80	2.96 ± 0.81	2.17 ± 0.85	2.34 ± 0.61
Qiang ethnic minority (*n* = 435)	2.43 ± 0.72	2.26 ± 0.86	3.01 ± 0.75	2.29 ± 0.86	2.51 ± 0.60
Tibetan (*n* = 356)	2.41 ± 0.72	2.23 ± 0.87	2.92 ± 0.75	2.24 ± 0.90	2.46 ± 0.62
Male (*n* = 326)	2.44 ± 0.80	2.23 ± 0.90	2.93 ± 0.82	2.28 ± 0.96	2.49 ± 0.69
Female (*n* = 615)	2.39 ± 0.67	2.21 ± 0.83	2.97 ± 0.73	2.24 ± 0.84	2.46 ± 0.57
Junior high (*n* = 238)	2.10 ± 0.72	1.97 ± 0.91	2.73 ± 0.80	2.00 ± 0.89	2.21 ± 0.63
Senior high (*n* = 703)	2.51 ± 0.69	2.30 ± 0.82	3.03 ± 0.73	2.34 ± 0.86	2.56 ± 0.58
Total	2.41 ± 0.72	2.21 ± 0.86	2.96 ± 0.76	2.26 ± 0.88	2.47 ± 0.61

Assuming a similar degree of harm during the COVID-19 pandemic and using the same survey tools, a study of 936 junior middle school students in Quanzhou City, Fujian Province, was used as a reference (Li and Li, 2015). The total average score and standard deviation of academic burnout were employed to conduct a summary independent samples t-test. The results showed that the degree of academic burnout among adolescents in Wenchuan was significantly higher than those in Quanzhou, Fujian Province [*t* (1875) = 9.96, *p* < 0.001].

Single factor analysis of variance and independent samples t-tests were used to test the ethnic, gender, and academic stage differences in academic burnout. The results showed no significant differences in ethnicity [*F* (2, 898) = 2.59, *p* > 0.05] and gender [*t* (939) = 0.50, *p* > 0.05] on academic burnout among adolescents in the Wenchuan area during the COVID-19 pandemic. The academic burnout of junior high school students was significantly lower than that of high school students [*t* (939) = −8.00, *p* < 0.001].

A repeated-measures analysis of variance was conducted to compare the four dimensions of academic burnout. The results indicated significant differences in the scores among the dimensions [*F* (2.30, 2,820) = 2353.96, *p* < 0.001]. *Post hoc* comparisons showed that emotional exhaustion was significantly higher than the other three dimensions (*p* < 0.001), and academic inefficiency was significantly higher than physical exhaustion and alienation between teachers and students (*p* < 0.001). There was no significant difference in physical exhaustion and alienation between teachers and students (*p* > 0.05).

### Relationship between PTG, academic burnout, and core belief challenge

Pearson’s correlation coefficients were calculated to examine the relationships between the main study variables (see Table 3). The categorical variable “academic stage” was dummy coded. The results showed a significant negative correlation between PTG and academic burnout among adolescents in the Wenchuan region. Furthermore, core belief challenge correlated positively with PTG and negatively with academic burnout.

**Table 3:** Descriptive statistics and inter-correlations between variables.

Variables	*M*	*SD*	1	2	3
1. Academic stage (junior high = 0; senior high = 1)	–	–	–		
2. PTG	2.43	1.20	−0.09^**^	–	
3. Academic burnout	2.47	0.61	0.25^***^	−0.18^***^	–
4. Core belief challenge	2.51	1.20	−0.05	0.43^***^	−0.10^**^

*N* = 941; ^*^p < 0.05; ^**^p < 0.01; ^***^p < 0.001.

Hierarchical regression analysis was conducted to test the moderating effect of core belief challenge on the relationship between PTG and academic burnout. The academic stage was also dummy coded and added as a control variable to the first-level regression. Centralized PTG and core belief challenge were added to the second-level regression. The interaction between PTG and core belief challenge was added to the third-level regression (Academic burnout was used as the outcome variable).

As reflected in Table 4, PTG has a significant negative predictive effect on academic burnout, while core belief challenge did not. The interaction between PTG and core belief challenge had a significant negative predictive effect on academic burnout. This indicated that core belief challenge played a significant moderating role between PTG and academic burnout. To further analyze the moderating effect of the core belief challenge, this study divided core belief challenge scores into three groups—high (M + 1SD, >3.71), medium (M, 1.31–3.71), and low (M − 1SD, <1.31) levels—for the simple effects analysis. The results are presented in Table 5.

**Table 4:** Moderating effect of the core belief challenge on PTG and academic burnout.

Variables	Academic burnout					
	Model 1		Model 2		Model 3	
	*β*	*SE*	*β*	*SE*	*β*	*SE*
Academic stage (junior high = 0; senior high = 1)	0.25^***^	0.94	0.24^***^	0.93	0.24^***^	0.92
PTG			−0.15^***^	0.02	−0.13^**^	0.02
Core belief challenge			−0.02	0.04	−0.04	0.04
PTG × core belief challenge					−0.11^**^	0.03
*R*	0.25		0.30		0.32	
*R* ^2^	0.06		0.09		0.10	
Δ*R*^2^	0.06		0.03		0.01	
*F*	63.99^***^		12.75^***^		11.87^**^	

^**^p < 0.01; ^***^p < 0.001.

**Table 5:** Influence of PTG on academic burnout at different levels of core belief challenge.

Core belief challenge	*β*	*SE*	*t*	*p*	Lower	Upper
*M*-1*SD*	−0.07	0.02	−0.71	0.48	−0.06	0.03
*M*	−0.15	0.02	−3.32	0.00	−0.09	−0.02
*M* + 1*SD*	−0.23	0.02	−4.97	0.00	−0.14	−0.06

**Figure 1:**
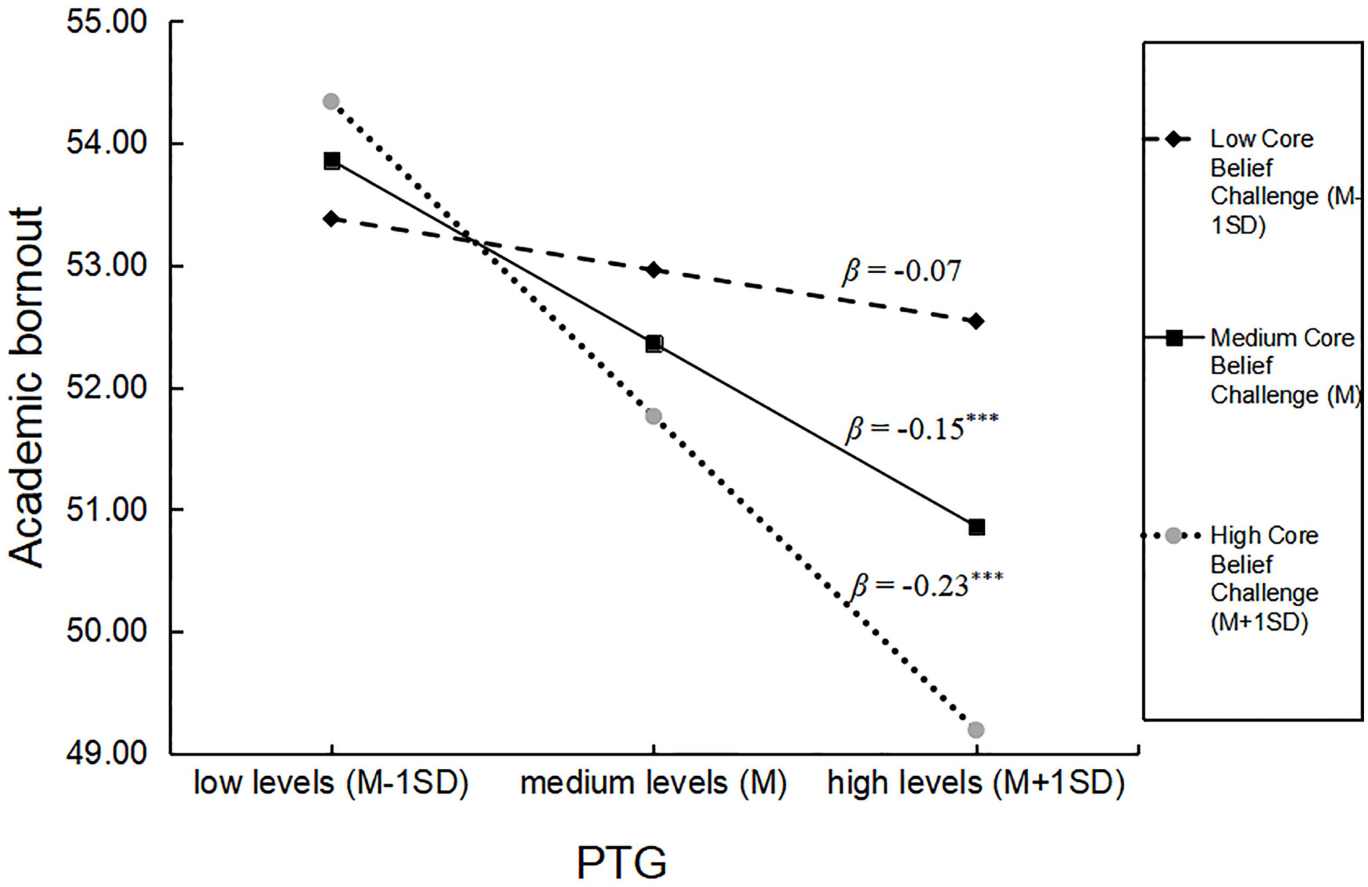
Moderating effect of the core belief challenge on the relationship between PTG and academic burnout in adolescents.

The effect of PTG on academic burnout was not significant for low core belief challenge (*β*_(M−1SD)_ = −0.07, *p* > 0.05). PTG had a significant negative predictive effect on academic burnout (*β*_(M)_ = −0.15, *β*_(M+1SD)_ = −0.23, *p* < 0.001) for medium and high levels of core belief challenge. The simple slope test diagram in Figure 1 illustrates the moderating effect of core belief challenge on the relationship between PTG and academic burnout.

## Discussion

### Characteristics of PTG among adolescents in Wenchuan area

This study found that five months after the outbreak of the COVID-19 pandemic, the level of PTG in adolescents in the Wenchuan area was relatively high, and its prevalence was above the moderate level (32.3%). Compared to previous studies on adolescents in Leshan City and Jianyang City of Sichuan Province (Tang et al., 2022), the prevalence and PTG levels in the Wenchuan area were significantly higher, supporting H1. This may be related to adolescents in Wenchuan being influenced more by Tibetan Buddhism and traditional Qiang culture (Ran, 2011; Li Z., 2019). Therefore, they may be more active and adaptive to traumatic events (Fan et al., 2009). However, unlike H1, this study found no significant ethnic or sex differences in PTG. This may be related to the high degree of ethnic integration in the Wenchuan area, where adolescents of different ethnicities and genders are widely affected by local cultural customs, and generally have higher PTG. We also found that junior high school students’ PTG was higher than that of senior high school students, supporting H1. This may be because junior high school students had lower anxiety about death (Wang et al., 2018) and a more positive outlook toward traumatic events during the COVID-19 pandemic (Huang and Yao, 2020).

Furthermore, we found that adolescents’ self-perception changed the most in all the PTG dimensions, which differs from the results of previous studies on the Wenchuan and Southeast Asian earthquakes (Tang, 2007; Taku et al., 2012; An et al., 2013). This may be because compared with regional natural disasters such as earthquakes, social attention, and assistance in Wenchuan after the outbreak were significantly lower than those after the earthquake. Affected by prevention and control requirements, there was more interpersonal isolation during the pandemic than the mutual cooperation seen after natural disasters. By contrast, home segregation and reduced social activities may have increased self-reflection and improved self-protection, thus increasing perceived changes of self.

### Characteristics of academic burnout among adolescents in Wenchuan area

This study found that the level of academic burnout among adolescents in the Wenchuan area was significantly higher than previous results in Quanzhou City, Fujian Province (Li and Li, 2015), supporting H1 to some extent. This is possibly related to the lack of medical resources in ethnic minority areas, relatively regressive public health services, and online learning conditions (Fang, 2021). Adolescents are more prone to anxiety and academic burnout. This study also found that academic burnout among junior high school students was significantly lower than that among senior high school students, which is consistent with previous studies (Sunawan et al., 2021) and H1. This may be due to the higher academic stress and anxiety among high school students during the pandemic. In addition, research has shown that junior high school students use more positive coping styles, such as problem solving, help-seeking, and rationalization (Wang and Ding, 2003). However, unlike H1, this study found no ethnic differences in academic burnout. This may be related to the high degree of ethnic integration in the Wenchuan region. Additionally, this may be because academic burnout is significantly affected by the situation. During the COVID-19 pandemic, the social and cultural environment, academic stress, and difficulties of adolescents from different ethnic groups in the region demonstrated more similarities than differences.

This study found that emotional exhaustion was the most serious dimension of academic burnout among adolescents in Wenchuan. This may be due to the subjective trauma exposure associated with the pandemic and increased anxiety, depression, and stress (Nicole et al., 2021). Furthermore, the increase in parent–child conflict (Liu et al., 2021) and decrease in social and outdoor activities (de Figueiredo et al., 2021) during isolation may have further exacerbated emotional exhaustion among adolescents.

### Relationship between PTG, academic burnout, and core belief challenge

This study found a significant negative correlation between PTG and academic burnout. The higher the PTG level of adolescents, the lower the level of academic burnout, which supports H2 and the results of previous studies (Li Y., 2019; An et al., 2022). Studies have shown that individuals with higher PTG are better at effective emotion regulation and obtain a higher level of positive emotions and self-efficacy (Mo et al., 2013), which may alleviate academic burnout among adolescents. Additionally, core belief challenge can significantly moderate the relationship between PTG and academic burnout, supporting H3. The results also support the broken assumptive worlds hypothesis (Janoff-Bulman, 1989) and the affective-cognitive processing model (Joseph et al., 2012) to some extent, suggesting that core cognitive change plays an important role in the process of PTG alleviating academic burnout.

When the core belief challenge level was above the medium level, PTG had a significant negative predictive effect on academic burnout. This result supports the view that PTG is adaptive (Tedeschi and Calhoun, 1996), meaning that the positive growth experienced by adolescents in the process of fighting against trauma can improve their learning attitudes and behaviors. Studies have shown that cognitive reconstruction caused by core belief challenge can change individual worldviews and perceptions of stressors (Joseph and Linley, 2005; Cho and Park, 2013). During the COVID-19 pandemic, higher core belief challenge may lead adolescents to rethink the value of life, adopt more positive coping styles, plan their own ideals, and live more actively, and resist the risk of increased academic burnout.

In contrast, when the level of core belief challenge was low, PTG had no significant predictive effect on academic burnout. According to the functional descriptive model of PTG (Tedeschi and Calhoun, 2004), positive changes after trauma require cognitive reconstruction. Adolescents with low core belief challenge may have less active thinking and cognitive reconstructions. PTG may enhance psychological adaptation and reduce negative emotions (Sumalla et al., 2009), but it is difficult to translate this into cognitive and behavioral changes.

## Contributions and implications

After the large-scale outbreak of the COVID-19 pandemic, how adolescents alleviate academic burnout and invest in and adapt to the new learning environment has become an important practical issue for educational researchers and practitioners. Based on the results of this study, we propose the following four recommendations:

First, considering the high level of academic burnout among adolescents in Wenchuan, it is necessary to strengthen the evaluation of adolescents’ psychological adaptation, particularly for high school students who need continuous psychological intervention. Considering serious emotional exhaustion, it is necessary to teach local adolescents skills in emotion regulation and coping with changes in learning conditions. Furthermore, a family school-society linkage is needed to enable adolescents to experience more interpersonal connections and social support while preventing and controlling the pandemic.

Second, it provides full play to the advantages of ethnic minority cultures in promoting PTG among local adolescents. It is necessary to fully tap and use unique ethnic mental health education resources in sports, dance, religious activities, and customs to promote adolescents’ self-experience and interpersonal cooperation as well as experience with philosophical wisdom, and help more adolescents achieve PTG.

Third, there should be focus on cognitive reconstruction and value guidance of adolescents during the pandemic. Based on the key role of core belief challenge between PTG and academic burnout, school mental health education should focus on guiding adolescents’ reflections on the pandemic and themselves. For example, it is necessary to encourage them to express their feelings and ideas about the impact of the pandemic, guide them to actively redefine trauma, think about its positive impact, and improve their patriotism to promote their cognitive reconstruction of pandemic trauma and prevent and reduce psychological and behavioral problems, such as academic burnout.

Fourth, it is necessary for the government to accelerate the construction of public psychological service system in ethnic areas during the COVID-19 pandemic. On the one hand, it is necessary to strengthen the promotion of existing hotline psychological anti-pandemic services and popular science propaganda in ethnic areas, so as to provide more targeted psychological counseling and health education for adolescents in ethnic areas. On the other hand, combined with the current situation that the extreme shortage of professional psychological teachers in ethnic minority areas and it is difficult to effectively carry out psychological screening and key population guidance during the pandemic, the education department could organize targeted teacher training and match assistance from relevant colleges and universities for ethnic minority areas.

## Limitations and future research

First, the cross-sectional research methods used in this study cannot reveal the dynamic changes in PTG, academic burnout, and their relationships among adolescents in the Wenchuan area over time. Thus, considering the persistent and repeated outbreak characteristics of the COVID-19 pandemic, it is necessary to conduct longitudinal studies in the future.

Second, this study did not directly sample the control group when comparing adolescents in Wenchuan and other regions using only previous studies as a reference. It failed to strictly control for the level of economic development, educational resources, and other irrelevant variables. Further studies should select areas with more similarities to conduct more rigorous comparative studies.

## Data availability statement

The original contributions presented in the study are included in the article/Supplementary material, further inquiries can be directed to the corresponding author.

## Ethics statement

Ethical review and approval was not required for the study on human participants in accordance with the local legislation and institutional requirements. Written informed consent to participate in this study was provided by the participants’ legal guardian/next of kin.

## Author contributions

XY, XW, and QC contributed to conception and design of the study. ZZ performed the statistical analysis and wrote the first draft of the manuscript. XY, ZZ, XW, and YG contributed to manuscript revision. All authors contributed to the article and approved the submitted version.

## Funding

This work was supported by Fundamental Research Funds for the Central Universities, Southwest Minzu University (2022SYB14) and Training Program for College Students' Innovation and Entrepreneurship, Southwest Minzu University (S202210656099).

## Conflict of interest

The authors declare that the research was conducted in the absence of any commercial or financial relationships that could be construed as a potential conflict of interest.

## Publisher’s note

All claims expressed in this article are solely those of the authors and do not necessarily represent those of their affiliated organizations, or those of the publisher, the editors and the reviewers. Any product that may be evaluated in this article, or claim that may be made by its manufacturer, is not guaranteed or endorsed by the publisher.

## Abbreviations

PTG: posttraumatic growth

## References

[ref1] Aljadani A. H. Ahmed A. Almehmadi S. Alhuwaydi A. Fathuldeen A. (2021). Epidemiology of burnout and its association with academic performance among medical students at hail university, Saudi Arabia. Sultan Qaboos Univ. Med. J. 21, e231–e236. doi: 10.18295/squmj.2021.21.02.011, PMID: 34221470 PMC8219327

[ref2] An Y. Huang J. Yeung E. T. F. Hou W. K. (2022). Academic burnout and posttraumatic growth predict trajectories of posttraumatic stress disorder symptoms of adolescents following Yancheng tornado in China. Int. J. Stress. Manag. 29, 143–153. doi: 10.1037/str0000240

[ref3] An Y. Y. Wu X. C. Liu C. H. Lin C. D. (2013). Neuroticism personality and posttraumatic growth in adolescent: the moderating effect of coping style and mediating effect of social support. Psychol. Dev. Educ. 29, 657–663. doi: 10.16187/j.cnki.issn1001-4918.2013.06.008

[ref4] Cann A. Calhoun L. G. Tedeschi R. G. Kilmer R. P. Gil-Rivas V. Vishnevsky T.. (2010). The core beliefs inventory: a brief measure of disruption in the assumptive world. Anxiety Stress Coping 23, 19–34. doi: 10.1080/10615800802573013, PMID: 19326274

[ref5] Carson J. (2001). The burnout companion to study and practice: A critical analysis. J. Occup. Organ. Psychol. 74, 111–112.

[ref6] Cho D. Park C. L. (2013). Growth following trauma: overview and current status. Terapia Psicológica 31, 69–79. doi: 10.4067/S0718-48082013000100007

[ref7] de Figueiredo C. S. Sandre P. C. Portugal L. C. L. Mázala-de-Oliveira T. da Silva Chagas L. Raony Í.. (2021). COVID-19 pandemic impact on children and adolescents' mental health: biological, environmental, and social factors. Prog. Neuro-Psychopharmacol. Biol. Psychiatry 106:110171. doi: 10.1016/j.pnpbp.2020.110171, PMID: 33186638 PMC7657035

[ref8] Dozois D. J. A. (2021). Anxiety and depression in Canada during the COVID-19 pandemic: A national survey. Can. Psychol. 62, 136–142. doi: 10.1037/cap0000251

[ref9] Erman Y. (2021). Posttraumatic growth and positive determinants in nursing students after COVID-19 alarm status: a descriptive cross-sectional study. Perspect. Psychiatr. Care 57, 1876–1887. doi: 10.1111/ppc.12761, PMID: [Epub ahead of print] 33728659 PMC8251109

[ref10] Fang J. W. (2021). The improvement of public health Services in Ethnic Minority Areas of China. J. Ethnol. 12, 33–40. doi: 10.3969/j.issn.1674-9391.2021.04.005

[ref11] Fan S. F. Yun G. L. Liu C. H. (2009). A brief review of psychological pain research: focused on the psychological pain of victims from the 5·12 earthquake. Adv. Psychol. Sci. 17, 631–638.

[ref12] Freudenberger H. J. (1974). Staff burn-out. J. Soc. Issues 30, 159–165. doi: 10.1111/j.1540-4560.1974.tb00706.x

[ref13] Gibbons S. Murphy D. Joseph S. (2011). Countertransference and positive growth in social workers. J. Soc. Work. Pract. 25, 17–30. doi: 10.1080/02650530903579246

[ref14] Gonzalez-Ramirez J. Mulqueen K. Zealand R. Silverstein S. Reina C. BuShell S.. (2021). Emergency online learning: college students' perceptions during the COVID-19 pandemic. Coll. Stud. J. 55, 29–46.

[ref15] Guessoum S. B. Lachal J. Radjack R. Carretier E. Minassian S. Benoit L.. (2020). Adolescent psychiatric disorders during the COVID-19 pandemic and lockdown. Psychiatry Res. 291:113264. doi: 10.1016/j.psychres.2020.113264, PMID: 32622172 PMC7323662

[ref16] Huang F. Yao B. X. (2020). Studying on the psychological crisis of death anxiety in the NCP epidemics and its countermeasures. Chin. Health Serv. Manag. 37, 778–781.

[ref17] Hu Q. Dai C. (2007). A research on middle school students’ learning burnout structure. Psychol. Sci. 30, 162–164. doi: 10.16719/j.cnki.1671-6981.2007.01.041

[ref18] Ickovics J. R. Meade C. S. Kershaw T. S. Milan S. Lewis J. B. Ethier K. A. (2006). Urban teens: trauma, posttraumatic growth, and emotional distress among female adolescents. J. Consult. Clin. Psychol. 74, 841–850. doi: 10.1037/0022-006X.74.5.841, PMID: 17032088

[ref19] Janoff-Bulman R. (1989). Assumptive worlds and the stress of traumatic events: applications of the schema construct. Soc. Cogn. 7, 113–136. doi: 10.1521/soco.1989.7.2.113

[ref20] Janoff-Bulman R. (2006). “Schema-change perspectives on posttraumatic growth” in Handbook of posttraumatic growth: research & practice. eds. Calhoun L. G. Tedeschi R. G. (Mahwah, NJ: Lawrence Erlbaum Associates Publishers), 81–99.

[ref21] Jian Y. Hu T. Zong Y. Tang W. (2022). Relationship between post-traumatic disorder and posttraumatic growth in covid-19 home-confined adolescents: the moderating role of self-efficacy. Curr. Psychol. J. Divers. Perspect. Divers. Psychol. Issues 2022, 1–10. doi: 10.1007/s12144-021-02515-8, PMID: 35018083 PMC8736319

[ref22] Joseph S. Linley P. A. (2005). Positive adjustment to threatening events: An organismic valuing theory of growth through adversity. Rev. Gen. Psychol. 9, 262–280. doi: 10.1037/1089-2680.9.3.262

[ref23] Joseph S. Murphy D. Regel S. (2012). An affective–cognitive processing model of post-traumatic growth. Clin. Psychol. Psychother. 19, 316–325. doi: 10.1002/cpp.1798, PMID: 22610981

[ref24] Kesimci A. Göral F. S. Gençöz T. (2005). Determinants of stress-related growth: gender, stressfulness of the event, and coping strategies. Curr. Psychol. 24, 68–75. doi: 10.1007/s12144-005-1005-x

[ref25] Kristo E. A. (2021). Post-traumatic growth among high school students during the COVID-19 pandemic: a survey study. Creat. Educ. 12, 1600–1607. doi: 10.4236/CE.2021.127121

[ref26] Lan L. Y. (2011). On the psychological regulation function of Tibetan Buddhism and post-disaster psychological crisis intervention. Relig. Stud. 3, 269–273.

[ref27] Li J. T. Li H. J. (2015). Relationship between peer interaction and learning burnout of junior middle school students in Quanzhou. Chin. J. Sch. Health 36, 1879–1881. doi: 10.16835/j.cnki.1000-9817.2015.12.040

[ref28] Li J. Yang Z. Qiu H. Wang Y. Jian L. Ji J.. (2020). Anxiety and depression among general population in China at the peak of the COVID-19 epidemic. World Psychiatry 19, 249–250. doi: 10.1002/wps.20758, PMID: 32394560 PMC7214959

[ref29] Li Q. Luo R. L. Zhang X. Y. Meng G. T. Dai B. B. Liu X. (2021). Intolerance of COVID-19-related uncertainty and negative emotions among Chinese adolescents: A moderated mediation model of risk perception, social exclusion and perceived efficacy. Int. J. Environ. Res. Public Health 18, 2864–2866. doi: 10.3390/ijerph18062864, PMID: 33799731 PMC8002157

[ref30] Li Y. (2019). Study on the development trajectory of posttraumatic stress response in post-disaster adolescent. master’s thesis. Nanjing: Nanjing Normal University.

[ref31] Li Z. Y. (2019). On the factors of Bonism in Qiang folk religion. Relig. Stud. 2, 179–183.

[ref32] Lin C. D. Wu X. C. Zhang Y. D. Zang W. W. Zhou X. Dai Y. (2013). Investigation on mental health state of primary and secondary school students after 30 months of Wenchuan earthquake. Psychol. Dev. Educ. 29, 631–640. doi: 10.16187/j.cnki.issn1001-4918.2013.06.005

[ref33] Liu J. Zhou T. Yuan M. Ren H. Bian X. Coplan R. J. (2021). Daily routines, parent–child conflict, and psychological maladjustment among Chinese children and adolescents during the COVID-19 pandemic. J. Fam. Psychol. 35, 1077–1085. doi: 10.1037/fam0000914, PMID: 34472935

[ref34] Mattson E. James L. Engdahl B. (2018). Personality factors and their impact on PTSD and post-traumatic growth is mediated by coping style among OIF/OEF veterans. Mil. Med. 183, e475–e480. doi: 10.1093/milmed/usx201, PMID: 29590428

[ref35] Milam J. Ritt-Olson A. Tan S. Unger J. Nezami E. (2005). The September 11th 2001 terrorist attacks and reports of posttraumatic growth among a multi-ethnic sample of adolescents. Traumatology 11, 233–246. doi: 10.1177/153476560501100404

[ref36] Mo K. Tang T. Cheng N. Yu Y. J. Peng L. Li M. (2013). The relationships among posttraumatic growth, affect and emotion regulation and self-efficacy in cancer patients. Chin. J. Nurs. 29, 143–153. doi: 10.1037/str0000240

[ref37] Nicole R. Anne M. B. Cooke J. E. Rachel E. Jenney Z. Sheri M. (2021). Global prevalence of depressive and anxiety symptoms in children and adolescents during COVID-19: A meta-analysis. JAMA Pediatr. 175:1142. doi: 10.1001/JAMAPEDIATRICS.2021.2482, [Epub ahead of print] 34369987 PMC8353576

[ref38] Ran R. G. (2011). On Qiang culture in Wenchuan. J. Chin. Cult. 5, 66–72.

[ref39] Salmela-Aro K. Upadyaya K. (2014). School burnout and engagement in the context of demands-resources model. Br. J. Educ. Psychol. 84, 137–151. doi: 10.1111/bjep.12018, PMID: 24547758

[ref40] Schaufeli W. B. Martínez I. M. Marques Pinto A. Salanova M. Bakker A. B. (2002). Burnout and engagement in university students: A cross-national study. J. Cross-Cult. Psychol. 33, 464–481. doi: 10.1177/0022022102033005003

[ref41] Schroevers M. J. Teo I. (2008). The report of posttraumatic growth in Malaysian cancer patients: relationships with psychological distress and coping strategies. Psycho-Oncology 17, 1239–1246. doi: 10.1002/pon.1366, PMID: 18457342

[ref42] Sumalla E. C. Ochoa C. Blanco I. (2009). Posttraumatic growth in cancer: reality or illusion? Clin. Psychol. Rev. 29, 24–33. doi: 10.1016/j.cpr.2008.09.006 18996633

[ref43] Sunawan S. Amin Z.N. Hafina A. Kholili M. (2021). “The differences of students’ burnout from level of education and duration daily online learning during COVID-19 pandemics,” in Proceedings of the International Conference on Industrial Engineering and Operations Management, 3723–3729.

[ref44] Taku K. Kilmer R. P. Cann A. Tedeschi R. G. Calhoun L. G. (2012). Exploring posttraumatic growth in Japanese youth. Psychol. Trauma Theory Res. Pract. Policy 4, 411–419. doi: 10.1037/a0024363

[ref45] Tang C. S. (2006). Positive and negative postdisaster psychological adjustment among adult survivors of the southeast Asian earthquake-tsunami. J. Psychosom. Res. 61, 699–705. doi: 10.1016/j.jpsychores.2006.07.014, PMID: 17084149

[ref46] Tang C. S. (2007). Posttraumatic growth of southeast Asian survivors with physical injuries: six months after the 2004 southeast Asian earthquake-tsunami. Australas. J. Disaster Trauma Stud. 2007-1. doi: 10.1037/t03776-000

[ref47] Tang W. Yan Z. Lu Y. Xu J. (2022). Prospective examination of adolescent emotional intelligence and post-traumatic growth during and after COVID-19 lockdown. J. Affect. Disord. 309, 368–374. doi: 10.1016/j.jad.2022.04.129, PMID: 35472475 PMC9035660

[ref48] Tedeschi R. G. Calhoun L. G. (1996). The posttraumatic growth inventory: measuring the positive legacy of trauma. J. Trauma. Stress. 9, 455–471. doi: 10.1002/jts.2490090305, PMID: 8827649

[ref49] Tedeschi R. G. Calhoun L. G. (2004). Posttraumatic growth: conceptual foundations and empirical evidence. Psychol. Inq. 15, 1–18. doi: 10.1207/s15327965pli150101

[ref50] Tomaszek K. Muchacka-Cymerman A. (2022). Student burnout and PTSD symptoms: the role of existential anxiety and academic fears on students during the COVID-19 pandemic. Depress. Res. Treat. 2022:9. doi: 10.1155/2022/6979310, PMID: 35096425 PMC8796705

[ref51] Ulset V. S. Soest T. (2022). Posttraumatic growth during the covid-19 lockdown: a large-scale population-based study among Norwegian adolescents. J. Trauma. Stress. 35, 941–954. doi: 10.1002/jts.22801, PMID: [Epub ahead of print] 35182076 PMC9305897

[ref52] Vaughn A. A. Roesch S. C. Aldridge A. A. (2009). Stress-related growth in racial/ethnic minority adolescents: measurement structure and validity. Educ. Psychol. Meas. 69, 131–145. doi: 10.1177/0013164408318775

[ref53] Wang C. Y. Pan R. Y. Wan X. Y. Tan Y. L. Xu L. K. Ho C. S.. (2020). Immediate psychological responses and associated factors during the initial stage of the 2019 coronavirus disease (COVID-19) epidemic among the general population in China. Int. J. Environ. Res. Public Health 17:1729. doi: 10.3390/ijerph17051729, PMID: 32155789 PMC7084952

[ref54] Wang G. H. Zhang Y. T. Zhao J. Zhang J. Jiang F. (2020). Mitigate the effects of home confinement on children during the COVID-19 outbreak. Lancet 395, 945–947. doi: 10.1016/s0140-6736(20)30547-x, PMID: 32145186 PMC7124694

[ref55] Wang J. Bu L. Li Y. Song J. Li N. (2021). The mediating effect of academic engagement between psychological capital and academic burnout among nursing students during the COVID-19 pandemic: a cross-sectional study. Nurse Educ. Today 102:104938. doi: 10.1016/j.nedt.2021.104938, PMID: 33934039 PMC8669342

[ref56] Wang J. S. Ding X. H. (2003). Research on the relationship between subjective well-being and coping style of junior middle school students. Chin. J. Public Health 10, 33–34.

[ref57] Wang X. B. Wang J. H. Cheng X. (2018). Influencing factorial analysis of death anxiety among middle school students in Guizhou Province. Chin. J. School Health 39, 1001–1003. doi: 10.16835/j.cnki.1000-9817.2018.07.012

[ref58] Wu X. Kaminga A. C. Dai W. Deng J. Wang Z. Pan X.. (2019). The prevalence of moderate-to-high posttraumatic growth: a systematic review and meta-analysis. J. Affect. Disord. 243, 408–415. doi: 10.1016/j.jad.2018.09.023, PMID: 30268956

[ref59] Xiong J. Lipsitz O. Nasri F. Lui L. M. W. Gill H. Phan L.. (2020). Impact of COVID-19 pandemic on mental health in the general population: a systematic review. J. Affect. Disord. 277, 55–64. doi: 10.1016/j.jad.2020.08.001, PMID: 32799105 PMC7413844

[ref60] Xu J. Liao Q. (2011). Prevalence and predictors of posttraumatic growth among adult survivors one year following 2008 Sichuan earthquake. J. Affect. Disord. 133, 274–280. doi: 10.1016/j.jad.2011.03.034, PMID: 21684612

[ref61] Yang F. Lin M. Y. Qian M. Y. (2010). A study of the relationship between posttraumatic growth and social support in children and adolescents following Wenchuan earthquake. Chin. J. Clin. Psych. 18, 614–617. doi: 10.16128/j.cnki.1005-3611.2010.05.027

[ref62] Ying L. Wang Y. Lin C. Chen C. (2016). Trait resilience moderated the relationships between PTG and adolescent academic burnout in a post-disaster context. Personal. Individ. Differ. 90, 108–112. doi: 10.1016/j.paid.2015.10.048

[ref63] Yuan G. Xu W. Liu Z. Liu C. Li W. An Y. (2018). Dispositional mindfulness, posttraumatic stress disorder symptoms and academic burnout in Chinese adolescents following a tornado: the role of mediation through regulatory emotional self-efficacy. J. Aggress. Maltreat. Trauma 27, 487–504. doi: 10.1080/10926771.2018.1433258

[ref64] Zhang D. Tian Y. X. Wu X. C. (2020). Public health model of psychological trauma prevention and intervention and its enlightenment. J. South China Norm. Univ. (Soc. Sci. Ed.) 4, 31–41.

[ref65] Zhang J. Yuan L. H. Lu X. H. Xiao Y. Liu Q. Zhang Q. X.. (2021). Effects of subjective trauma exposure on post-traumatic stress disorder in adolescents during COVID-19 outbreak: a moderated mediating model. Chin. J. Clin. Psych. 29, 748–752. doi: 10.16128/j.cnki.1005-3611.2021.04.017

[ref66] Zhen B. Yao B. Zhou X. (2022). How does parent–child communication affects posttraumatic stress disorder and growth in adolescents during the COVID-19 pandemic? The mediating roles of self-compassion and disclosure. J. Affect. Disord. 306, 1–8. doi: 10.1016/j.jad.2022.03.029, PMID: 35301037 PMC8920085

[ref67] Zhen R. Zhou X. (2021). Latent patterns of posttraumatic stress symptoms, depression, and posttraumatic growth among adolescents during the COVID-19 pandemic. J. Trauma. Stress. 35, 197–209. doi: 10.1002/jts.22720, PMID: [Epub ahead of print] 34339577 PMC8426724

[ref68] Zheng J. Morstead T. Sin N. Klaiber P. Umberson D. Kamble S.. (2021). Psychological distress in North America during COVID-19: the role of pandemic-related stressors. Soc. Sci. Med. 270:113687. doi: 10.1016/j.socscimed.2021.113687, PMID: 33465600 PMC9757831

[ref69] Zhou S. J. Zhang L. G. Wang L. L. Guo Z. C. Wang J. Q. Chen J. C.. (2020). Prevalence and socio-demographic correlates of psychological health problems in Chinese adolescents during the outbreak of COVID-19. Eur. Child Adolesc. Psychiatry 29, 749–758. doi: 10.1007/s00787-020-01541-4, PMID: 32363492 PMC7196181

[ref70] Zhou X. Wu X. C. An Y. Y. Cheng J. L. (2014). The roles of rumination and social support in the associations between Core belief challenge and post-traumatic growth among adolescent survivors after the Wenchuan earthquake. Acta Psychol. Sin. 46, 1509–1520. doi: 10.3724/sp.j.1041.2014.01509

[ref71] Zhou X. Wu X. Fu F. An Y. (2015). Core belief challenge and rumination as predictors of PTSD and PTG among adolescent survivors of the Wenchuan earthquake. Psychol. Trauma Theory Res. Pract. Policy 7, 391–397. doi: 10.1037/tra0000031, PMID: 25793513

[ref72] Zhou X. Zhen R. Wu X. (2017). Posttraumatic stress disorder symptom severity and control beliefs as the predictors of academic burnout amongst adolescents following the Wenchuan earthquake. Eur. J. Psychotraumatol. 8:1412227. doi: 10.1080/20008198.2017.1412227, PMID: 29296242 PMC5738653

[ref73] Zhou X. Zhen R. Wu X. (2019). Trajectories of academic burnout in adolescents after the Wenchuan earthquake: a latent growth mixture model analysis: corrigendum. Sch. Psychol. Int. 40:543. doi: 10.1177/0143034319866278

[ref74] Zis P. Artemiadis A. Bargiotas P. Nteveros A. Hadjigeorgiou G. M. (2021). Medical studies during the COVID-19 pandemic: the impact of digital learning on medical students’ burnout and mental health. Int. J. Environ. Res. Public Health 18:349. doi: 10.3390/ijerph18010349, PMID: 33466459 PMC7796433

[ref75] Žuljević M. F. Jeličić K. Viđak M. Đogaš V. Buljan I. (2021). Impact of the first COVID-19 lockdown on study satisfaction and burnout in medical students in Split, Croatia: A cross-sectional presurvey and postsurvey. BMJ Open 11:e049590. doi: 10.1136/bmjopen-2021-049590, PMID: 34187830 PMC8245286

