# Supplementary material for: Core belief challenge moderated the relationship between posttraumatic growth and adolescent academic burnout in Wenchuan area during the COVID-19 pandemic
Source: Front Psychol. 2022 Sep 28;13:1005176. doi: 10.3389/fpsyg.2022.1005176 (PMC9554300; doi:10.3389/fpsyg.2022.1005176)
Supplement: Supplementary file 2 [file Table_1.docx]

创伤后成长问卷（Posttraumatic Growth Inventory）

在**新冠肺炎疫情发生以来**，您发生了哪些变化，请仔细阅读各项问题并根据自己的感受，选择相应的选项。

| 题项 | 没有变化 | 变化很小 | 变化较小 | 变化一般 | 变化较大 | 变化很大 |
| --- | --- | --- | --- | --- | --- | --- |
| 1.我更懂得生命中哪些事情对我来说更重要 | 0 | 1 | 2 | 3 | 4 | 5 |
| 2.我更愿意尝试去改变那些需要改变的事情 | 0 | 1 | 2 | 3 | 4 | 5 |
| 3.欣赏我自己生命的价值 | 0 | 1 | 2 | 3 | 4 | 5 |
| 4.我发现在生活中遇到问题时，我可以靠自己的力量去解决 | 0 | 1 | 2 | 3 | 4 | 5 |
| 5.更能够理解某些神秘的事情（如宗教、神灵、 命运之神等） | 0 | 1 | 2 | 3 | 4 | 5 |
| 6.在遇到麻烦的时候，我可以找别人求助 | 0 | 1 | 2 | 3 | 4 | 5 |
| 7.感觉和别人更加亲近 | 0 | 1 | 2 | 3 | 4 | 5 |
| 8.到问题的时候，知道自己能够处理 | 0 | 1 | 2 | 3 | 4 | 5 |
| 9.愿意向他人诉说自己的情绪 | 0 | 1 | 2 | 3 | 4 | 5 |
| 10.能够接受事情自然发展的方式（顺其自然） | 0 | 1 | 2 | 3 | 4 | 5 |
| 11.珍惜每一天 | 0 | 1 | 2 | 3 | 4 | 5 |
| 12.能够理解别人的痛苦 | 0 | 1 | 2 | 3 | 4 | 5 |
| 13.在活着的时候，我应该把事情做得更好，不留下遗憾 | 0 | 1 | 2 | 3 | 4 | 5 |
| 14.我的生活中出现了以前根本不可能出现的机会 | 0 | 1 | 2 | 3 | 4 | 5 |
| 15.重视自己的人际关系 | 0 | 1 | 2 | 3 | 4 | 5 |
| 16.更加理解信仰宗教的行为 | 0 | 1 | 2 | 3 | 4 | 5 |
| 17.我发现我比自己想象的要坚强 | 0 | 1 | 2 | 3 | 4 | 5 |
| 18.我懂得了人是多么的美好 | 0 | 1 | 2 | 3 | 4 | 5 |
| 19.我培养了新的兴趣 | 0 | 1 | 2 | 3 | 4 | 5 |
| 20.知道自己在有些情况下是需要他人的，并且能够接受这一事实 | 0 | 1 | 2 | 3 | 4 | 5 |
| 21.我为自己的人生寻找到了新的道路 | 0 | 1 | 2 | 3 | 4 | 5 |
| 22.更加理解冥冥之中有某种不可控的力量存在 | 0 | 1 | 2 | 3 | 4 | 5 |

学业倦怠问卷（Adolescent Academic Burnout Inventory）

请根据自身与各项目所描述情况相符合的程度，选择相应的选项，请根据您的真实情况填写。

| 题项 | 从未如此 | 很少如此 | 有时如此 | 经常如此 | 总是如此 |
| --- | --- | --- | --- | --- | --- |
| 1.学习让我精神愉悦 | 1 | 2 | 3 | 4 | 5 |
| 2.每次考试我总有一种应付一下的感觉 | 1 | 2 | 3 | 4 | 5 |
| 3.我不信任老师的话。 | 1 | 2 | 3 | 4 | 5 |
| 4.我很少温习功课。 | 1 | 2 | 3 | 4 | 5 |
| 5.读书能够使我获得他人的尊重 | 1 | 2 | 3 | 4 | 5 |
| 6.我的睡眠越来越差 | 1 | 2 | 3 | 4 | 5 |
| 7.对于作业，即使我努力思考还是不会做 | 1 | 2 | 3 | 4 | 5 |
| 8.老师对我有某种成见，总是看我不顺眼 | 1 | 2 | 3 | 4 | 5 |
| 9.学习让我感到身体不舒服 | 1 | 2 | 3 | 4 | 5 |
| 10.我觉得老师把分数看得比人重要 | 1 | 2 | 3 | 4 | 5 |
| 11.当我读书时就忘记了一切烦恼 | 1 | 2 | 3 | 4 | 5 |
| 12.我的学习问题很多，但是我越来越无所谓 | 1 | 2 | 3 | 4 | 5 |
| 13.这段时间，总是觉得这里或者那里不舒服 | 1 | 2 | 3 | 4 | 5 |
| 14.我在学习上很懒散 | 1 | 2 | 3 | 4 | 5 |
| 15.我看到老师总是躲得远远的 | 1 | 2 | 3 | 4 | 5 |
| 16.我觉得学习能使我的潜能得到发挥 | 1 | 2 | 3 | 4 | 5 |
| 17.我的身体不如从前那么好了 | 1 | 2 | 3 | 4 | 5 |
| 18.作业做不完，我已经无所谓了 | 1 | 2 | 3 | 4 | 5 |
| 19.我上课老是打瞌睡 | 1 | 2 | 3 | 4 | 5 |
| 20.我已经习惯了考试成绩差 | 1 | 2 | 3 | 4 | 5 |
| 21.我想到今天学习的很多知识就感到心情愉悦 | 1 | 2 | 3 | 4 | 5 |

核心信念问卷（Core Beliefs Inventory）

请你想一下，在经历新冠肺炎疫情之后，你在多大程度上经历了以下题目提及的情况。（注意：以下题目中的此事特指新冠肺炎疫情）

| 题项 | 从未 | 偶尔 | 有时候 | 经常 | 大多数时候 | 总是 |
| --- | --- | --- | --- | --- | --- | --- |
| 1.经过此事，我会去想人们的经历是否公平、平等 | 0 | 1 | 2 | 3 | 4 | 5 |
| 2.经过此事，我会去想在自己身边发生的事情是不是可以“控制” | 0 | 1 | 2 | 3 | 4 | 5 |
| 3.经过此事，我学会去想别人思考、行动的原因 | 0 | 1 | 2 | 3 | 4 | 5 |
| 4.经过此事，我会重新思考自己对人际关系的看法 | 0 | 1 | 2 | 3 | 4 | 5 |
| 5.经过此事，我会重新思考自己对自身能力的看法，包括优势和弱势 | 0 | 1 | 2 | 3 | 4 | 5 |
| 6.经过此事，我会重新思考自己对未来的想法 | 0 | 1 | 2 | 3 | 4 | 5 |
| 7.经过此事，我会去思考自己对未来的想法 | 0 | 1 | 2 | 3 | 4 | 5 |
| 8.经过此事，我会重新思考自己的信仰 | 0 | 1 | 2 | 3 | 4 | 5 |
| 9.经过此事，我会去想自己作为一个“人”的价值意义 | 0 | 1 | 2 | 3 | 4 | 5 |
